# Disseminated Histoplasmosis in Persons with HIV/AIDS, Southern Brazil, 2010–2019

**DOI:** 10.3201/eid2803.212150

**Published:** 2022-03

**Authors:** Rossana Patricia Basso, Vanice Rodrigues Poester, Jéssica Louise Benelli, David A. Stevens, Melissa Orzechowski Xavier

**Affiliations:** Programa de Pós Graduação em Ciências da Saúde, Universidade Federal do Rio Grande, Rio Grande, Brazil (R.P. Basso, V.R. Poester, J.L. Benelli, M.O. Xavier);; Hospital Universitário Dr. Miguel Riet Corrêa Jr., vinculado à Empresa Brasileira de Serviços Hospitalares, Rio Grande (R.P. Basso, J.L. Benelli);; California Institute for Medical Research, San Jose, California, USA (D.A. Stevens);; Stanford University Medical School, Stanford, California, USA (D.A. Stevens)

**Keywords:** histoplasmosis, *Histoplasma capsulatum*, HIV/AIDS and other retroviruses, mycoses, pulmonary disease, tuberculosis and other mycobacteria, Brazil, bacteria

## Abstract

We evaluated disseminated histoplasmosis (DH) in HIV patients over 10 years in southern Brazil. The incidence was 12 cases/1,000 hospitalizations (2010–2019); the mortality rate was 35%. Tuberculosis frequently obscured the diagnosis of DH. We emphasize the need in our region to suspect and investigate DH using more sensitive methods.

Disseminated histoplasmosis (DH) is an AIDS-defining disease and one of the major causes of death in persons living with HIV/AIDS (PLHIV) (mortality rate ranging from 13% to 48%) (*1*–*4*). DH is a neglected disease because of its nonspecific symptoms, frequent misdiagnosis as tuberculosis (TB), and limited access to sensitive diagnostic methods (*3*,*5*).

Worsening this scenario, an epidemic of AIDS is underway in Brazil; >800,000 new cases have been diagnosed in recent decades (*6*). Therefore, efforts are necessary to understand the epidemiology of DH/AIDS co-infection in the areas to which these diseases are endemic. We evaluated the clinical and epidemiologic profile of patients with DH/AIDS co-infection in a reference service for PLHIV over 10 years in southern Brazil and compared the incidence in periods before and after an internal hospital improvement of DH investigation.

## The Study

We performed a retrospective study including all DH cases diagnosed in persons with HIV/AIDS at a regional reference service in University Hospital Dr. Miguel Riet Corrêa Jr. (UH-FURG-Ebserh), a 207-bed tertiary hospital in Rio Grande, Brazil, that serves as reference center for 21 cities in Brazil. The hospital has an average of 257 HIV/AIDS hospitalizations/year (*7*). DH cases were defined by 1) classical methods: growth of *H. capsulatum* in culture, presence of blastoconidia suggestive of *H. capsulatum* by Gomori-Grocott stain (direct mycological examination or histopathology), or both; 2) serologic method: positive immunodiffusion test (IMMY, https://www.immy.com); or 3) urinary antigen test: positive immunoenzymatic assay (IMMY). Patients with clinical suspicion of DH and >1 of these diagnostic criteria were included. The study was approved by our university ethics committee (CEP/FURG, approval no. 234/2018).

We analyzed databases from the hospital for clinical and epidemiologic evaluation. In cases in which HIV and AIDS were diagnosed simultaneously, DH was considered the AIDS-defining illness. We calculated the overall incidence rate of DH per 1,000 hospitalizations of persons with AIDS at UH-FURG-Ebserh (*8*); we then compared that with rates before improvement of DH investigation (2010–2016) and after improvement of DH investigation (2017–2019). These improvements consisted of health education and training of health professionals to improve clinical suspicion and implementing urinary antigen detection as another diagnostic method. Descriptive and frequencies analyses were performed in SPSS Statistics 25.0 (IBM, https://www.ibm.com).

Our study included 31 cases of DH, representing an overall incidence of 12 new cases/1,000 PLHIV hospitalized at UH-FURG-Ebserh. In the first period (2010–2016), 15 cases were diagnosed in 7 years, a rate of 8/1,000 hospitalizations. After more sensitive testing and enhanced physician training were implemented, 16 cases were diagnosed in only 3 years (2017–2019), a rate of 24/1,000 hospitalizations, a substantial increase.

Most patients were men; mean age was 41 (range 21–61) years (Table 1). Except for 3, all had co-infections diagnosed concomitantly (Table 2). The use of antiretroviral therapy at time of DH diagnosis was irregular or nonexistent in 90% (n = 28) of patients. Only 4 had >200 cells/mm^3^ of CD4+ lymphocytes (mean 109 cells/mm^3^; range 7–752 cells/mm^3^). In 6 (19%), DH was the AIDS-defining illness. A total of 3 persons had DH associated with a systemic inflammatory response syndrome.

**Table 1:** Clinical-epidemiologic data of 31 disseminated histoplasmosis cases diagnosed in persons living with HIV/AIDS, University Hospital Dr. Miguel Riet Corrêa Jr., Rio Grande, Brazil, 2010–2019

Variable	Frequency, % (no./total no. participants)
M	74 (21/31)
F	26 (8/31)
Signs and symptoms	
Weight loss	100 (31/31)
Fever (>37.8°C)	100 (31/31)
Respiratory: cough and/or dyspnea	100 (31/31)
Cutaneous: papular and/or ulcerated	52 (16/31)
Neurologic: disorientation, focal deficit, paresthesia, confusion, headache and/or hemiplegia	52 (16/31)
Digestive: abdominal distension and pain, diarrhea and/or nausea	81 (25/31)
Hepatomegaly	55 (17/31)
Splenomegaly	81 (25/31)
Generalized lymph node enlargement	35 (11/31)
Image exams	
Interstitial lung pattern	55 (17/31)
Reticulonodular lung pattern	32 (10/31)
Pulmonary nodules	6 (2/31)
Mediastinal lymphadenopathy	26 (8/31)
Blood assays	
Anemia	100 (31/31)
Inflammatory marker*	100 (31/31)
Liver damage marker†	84 (26/31)
Tissue injury marker‡	87 (27/31)
Thrombocytopenia	74 (23/31)
HIV assays	
CD4+ lymphocytes ≤100/mm^3^	71 (22/31)
CD4+ lymphocytes ≤50/mm^3^	48 (15/31)
HIV Viral load ≥50,000 copies	90 (26/29)
First choice antifungal treatment	
None	3 (1/31)
Amphotericin B deoxycholate	81 (25/31)
Itraconazole	16 (5/31)
Outcome after 12 months	
Alive	65 (20/31)
Dead	35 (11/31)

*C-reactive protein increased. †Alkaline phosphatase increased. ‡Lactate dehydrogenase increased.

**Table 2:** Frequency of co-infections in 31 patients with disseminated histoplasmosis diagnosed in persons living with HIV/AIDS, University Hospital Dr. Miguel Riet Corrêa Jr., Rio Grande, Brazil, 2010–2019

Infectious disease	Frequency, % (no./total no. participants)
Oral candidiasis	61 (19/31)
Confirmed tuberculosis	29 (9/31)
Neurotoxoplasmosis	29 (9/31)
Pneumocystosis	23 (7/31)
Herpetic encephalitis	3 (1/31)
Herpes zoster	3 (1/31)
Syphilis	3 (1/31)
Medullary cytomegalovirus	3 (1/31)
*Mycobacterium avium* infection	3 (1/31)
Herpes simplex infection	3 (1/31)
Hepatitis C	3 (1/31)

Eight (26%) DH patients were empirically treated for TB (*9*); no cases were confirmed by GeneXpert MTB/RIF (Cepheid, https://www.cepheid.com). Up to 12 (mean 5) clinical samples/patient were submitted for TB investigations before suspicion of DH.

DH was diagnosed through classical mycologic exams in 14 (45%) patients, serologic tests in 9 (29%) patients, and urinary antigen assay in 4 (13%) patients. Four (13%) patients had >2 positive results by different methods (Figure). The diagnosis of histoplasmosis occurred after a mean of 10 (range 1–28) days from the beginning of hospitalization. This timing probably represents an underestimated delay, because several patients reported symptoms that could have led to a diagnostic workup before the illness progressed to a point at which hospitalization was required.

**Figure:**
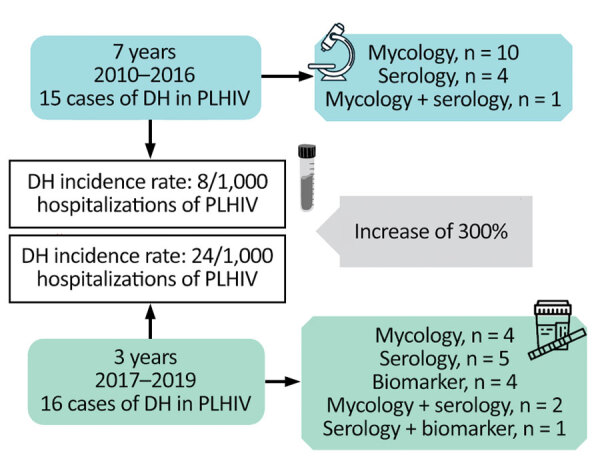
Approach used for the diagnosis of 31 cases of DH in PLHIV from a tertiary hospital in southern Brazil, 2010–2019. The incidence rate of DH between periods before (2010–2016) and after (2017–2019) implementation of the urinary antigen test shows an increase of 300%. DH, disseminated histoplasmosis; PLHIV, persons living with HIV.

The treatment of choice for 81% of patients was intravenous amphotericin deoxycholate (0.7–1 mg/kg/d, to a maximum 50 mg/d) for 14 days, followed by oral itraconazole (200 mg every 8 h for 3 days, then 200 mg every 12 h) for 12 months. A total of 5 (16%) patients were treated only with itraconazole (4 with early diagnosis of DH and 1 with renal dysfunction). Twelve months after the DH diagnosis, 35% of the patients had died (Table 1); 1 died before laboratory confirmation, and 4 died within an average of 25 (range 0–62) days after diagnosis of DH. Three patients died after 5–6 months while being treated with itraconazole, and 3 had recurrence of the disease after 6, 7, or 12 months because of antifungal interruption, which resulted in death (Table 1).

## Conclusions

DH causes severe clinical manifestations in PLHIV that can lead to death (*10*). Improved knowledge of the local epidemiology of DH and education of reference services for PLHIV are essential to reduce underdiagnosis and contribute to patient survival (*11*), especially in Rio Grande, a harbor city with the highest rate of HIV/AIDS among cities in Brazil with >100,000 inhabitants (*7*).

DH was the AIDS-defining illness in 21% of the patients in this study. Other co-infections (*12*,*13*) were noted. Respiratory signs, splenomegaly, and cutaneous lesions were more commonly described in our patients than in other studies, possibly because late diagnosis led to more severe extent of the disease (*12*,*13*). The high rate of neurologic impairment in our patients can be attributed to their co-infections; in 69% (9/13) of patients, this impairment was ascribed to neurotoxoplasmosis or herpetic encephalitis. In addition, neurologic signs were detected in 5 other patients without an ascribed neuropathogenic condition, meriting additional investigations.

Many of our patients were exhaustively investigated for TB, and 26% were empirically treated for TB despite negative results from the highly sensitive GeneXpert MTB/RIF assay (Cepheid). The long investigations for TB delayed the confirmation of the DH diagnosis despite descriptions of the concurrence of these 2 diseases (*14*). TB is a major opportunistic disease in PLHIV (*15*), evidenced by 29% of our DH patients with concomitant TB. Thus, the investigation of both diseases must occur simultaneously (*12*), and DH must be investigated in PLHIV with CD4+ lymphocytes <200 cells/mm^3^ (*11*).

Tests to detect *Histoplasma* antibodies have poor sensitivity (30%–70%) in immunocompromised patients (*1*). Diagnostic methods with high rates of sensitivity and specificity are vital in areas where histoplasmosis is endemic and could improve the likelihood of early diagnosis and favorable outcomes for patients (*1*,*12*). An improvement in the investigation of DH in PLHIV with respiratory symptoms occurred in the UH-FURG-Ebserh in 2017, through a collaboration in a multicenter study (*12*). Subsequently, the results contributed to the acquisition of the urinary antigen assay by UH-FURG-Ebserh. In the last 3 years of our study, the urinary antigen test was the only method able to detect 25% (4/15) of our patients with DH. The detection of urinary antigens is the standard for DH diagnosis in immunosuppressed patients (*11*).

The mortality rate in our series (35%) was similar to the rate described in a systematic review from Brazil histoplasmosis cases (33%) (*10*). Therefore, the underdiagnosis of DH in PLHIV is a national problem in Brazil that must be urgently changed. In our hospital, DH was responsible for high rates of illness in PLHIV, up to 24 cases/1,000 hospitalizations, and high mortality rates (35%). In addition, we emphasize that 29% of patients were co-infected with TB, a disease with symptoms overlapping with histoplasmosis. Simultaneous investigation for the 2 diseases in all PLHIV patients living in areas in which histoplasmosis is endemic is mandatory.
